# Factors associated with subjective state of health in college students

**DOI:** 10.3389/fpsyg.2022.985982

**Published:** 2022-10-13

**Authors:** Mahdi Rezapour

**Affiliations:** Independent Researcher, Marlborough, MA, United States

**Keywords:** subjective health, epidemic, student wellbeing, emotions, COVID-19

## Abstract

Although the COVID-19 pandemic has been deeply painful, it has provided a rare opportunity to study the behavioral responses of individuals in adapting to an unprecedented life event. An analysis of participants’ subjective health ratings during the COVID-19 pandemic was conducted by utilizing data from a survey of college students across seven universities in the US. In this study, we challenged the unidimensional factors to the subjective wellbeing by considering all multiplicative associations of those factors. Considering the interaction terms is especially important as not considering those impacts might obscure our understanding regarding the real associations. It was found that while higher screen hours, BMI, and various negative feelings are negatively associated with higher subjective health, higher family income, social class, and students’ and their mothers’ educations are associated with a higher subjective well-being. However, the impacts of the majority variables are interactive. For instance, the impact of mother’s education varies based on the genders of students, or the impact of screen hours differs based on family income. In addition, the degree students limit of exercise at home or gym changes based on the negative feeling they experience during the pandemic. Remarkably, during the pandemic while irrationally limiting exercise at home was associated with a lower subjective health, limiting exercise at gym was positively associated with the response.

## Introduction

The current COVID-19 pandemic has greatly affected peoples’ lives, influencing mental and physical health. It has generated an atmosphere of anxiety and depression and has disrupted travel plans, panic buying, social isolation, and media information overload (Ho et al., 2020). An aspect of mentality that may be affected especially by this type of unprecedented life event is the subjective wellbeing, which is a person’s evaluation of her or his own physical and emotional state.

The terms “health” and “wellbeing” have been used at times interchangeably (Law et al., 1998), so a brief clarification of the study’s focus is in order. Although subjective wellbeing is not necessarily linked to the absence of illness or developmental deficiencies, it is a fundamental facet of life quality that can be measured objectively or subjectively (Keyes, 2006). The subjective wellbeing reflects both physical and emotional wellbeing along with engagement in purposeful activities, suggesting that subjective wellbeing can reasonably be viewed as an overall evaluation of life quality from the individual’s perspective (Pavot et al., 2018).

Studying subjective health, in addition to objective health, is important because these two factors are associated with each other (Pinquart, 2001). Studies have been looked at the above associations from different perspectives. For instance, it has been discussed that subjective health has a stronger relationship with mental health than factors such as human capital or demographic characteristics (McKee-Ryan et al., 2005). In another study, the impacts of various life events such as divorce, unemployment, and retirement were investigated on cognitive wellbeing (Luhmann and Hawkley, 2016). The results highlighted that the impact of life events on cognitive wellbeing is consistent and strong.

Factors likely to be associated with both subjective and objective health include various emotional and behavioral characteristics. For instance, in evaluating determinants of health in students, it was found that the impact of trait emotional intelligence on general health is partially mediated by humor style (Greven et al., 2008). In addition, most important factors associated with the objective wellbeing of people could be summarized as education, income, physical health, personal security, and subjective wellbeing (Initiative, 2011).

However, there is an extant literature dealing with the subjective health, while focusing only on students and especially evaluating the measure during a dramatic life-changing event like COVID-19. In addition, the majority of previous studies considered the relationship between subjective health and other predictors to be additive. This study challenges the assumption in the literature review that the impacts of predictors on the subjective wellbeing are not necessarily additive.

For instance, does the associations between gender and subjective health is stable or varies based on various students,’ or their parents’ characteristics? Or does the associations between various negative feelings, such as being afraid, and subjective health varies based on students with different body mass index (BMI). Studying those associations is especially important at the time of the pandemic, as it provides a unique opportunity to study students’ behavioral and emotional feelings.

Here, the subjective health is treated as a multidimensional structure, which could be associated with various negative emotions and healthy and unhealthy behaviors. In this study, data from an earlier survey of college students from seven universities in the US during the COVID-19 pandemic (Browning et al., 2021) were examined with a focus on students’ ratings of their own health, with the aim of identifying factors relevant to those subjective evaluations. Studying subjective health is especially considered, as it associates with the objective health of students (Pinquart and Sorensen, 2001).

The main questions this study seeks to answer include:

1. Does parents’ education, social class, or wealth of students’ family has any impact on the subjective wellbeing perception?
2. Does doing exercise at home or gym is associated with varied level of subjective wellbeing?
3. Do the impacts of considered variables are unidimensional or varied based on other variables?

Again, the responses to the above questions are important due to the expected associations between subjective and objective health, which is consequently expected to impact the wellbeing of students.

## Data

The web-based survey was distributed across 14,174 students who enrolled in seven universities in the US, where 2,534 students who completed responses were obtained from the distributed questionnaires (Browning et al., 2021). It should be noted that the survey questions were collected where the US was experiencing a severe lockdown and social distancing. As is evident in Table 1, participants were asked about their demographic and individual characteristics, their emotions, and their behaviors. Question content is largely evident in Table 1, but additional information regarding a few items are provided here.

**TABLE 1:** Descriptive statistics of important predictors.

Coefficients	Average	Var.	Min.	Max.
General health: poor (1), average (2), good (3), very good (4), and excellent (5)	3.34	1.011	1	5
Gender, female (1) and male (0)	0.61	0.233	0	1
Irritability due to COVID-19, not at all (0) and extremely guilty (100)	59.43	791.258	0	100
Guilty due to COVID-19, not at all (0) and extremely guilty (100)	24.36	657.680	0	100
Screen hours, last 24 h	7.74	7.210	0	12
Relative family income: well below average (1), slightly below average (2), average (3), slightly above average (4), and well above average (5)	2.27	0.635	1	3
Limiting exercise at gym due to COVID-19, never (1), rarely (2), sometimes (3), most of the time (4), and always (5)	4.65	0.938	1	5
BMI, underweight	0.05	0.05	0	1
BMI, overweight	0.10	0.084	0	1
Feeling afraid due to COVID-19	50.54	741.890	0	100
Social class of a student, working class (1), lower middle-class (2), middle-class (3), upper middle-class (4), and upper class (5)	2.82	1.040	1	5
Student education achievement, graduate (1) and undergrad (0)	0.20	0.158	0	1
Limiting exercise at home due to COVID-19, never (1), rarely (2), sometimes (3), most of the time (4), and always (5)	2.32	1.538	1	5
Limiting outdoor activities due to COVID-19, never (1), rarely (2), sometimes (3), most of the time (4), always (5)	3.15	1.730	1	5
Worry due to COVID-19, not at all (0) and extremely guilty (100)	4.02	2.760	1	7

Negative emotions, such as being afraid, irritable, guilty, and sad, were based on the development of the positive and negative affect schedule (Watson and Clark, 1994). Participant burden was minimized by using items answered on the visual analog scale (VAS) (Jiang et al., 2016). These questions are related to how much time students thought about the pandemic (a concept derived from eating disorder literature) (Babbitt et al., 1990).

Different risk factors, such as gender, age, perceived social class, race, academic status, parental education, and relative family income, were incorporated in the survey. Health, in general, from poor to excellent (The Centers for Disease Control and Prevention’s [CDC], 2018), and BMI were measured as potential-confounding factors (Xiao et al., 2020). Time spent on screens or performing exercise was included as possible lifestyle-related factors (Tudor-Locke et al., 2010).

Participants were asked how much time they spent in front of screens daily, including smartphone/computer, watching television, or online gaming. To respond, participants could slide a bar to the right or left enabling them to answer in partial hours with decimal places. Participants were also asked about various seemingly rational behaviors, such as limiting exercise at gym (during the pandemic), and irrational behaviors, such as limiting exercise at home. Consideration of those factors was especially important to explore changes in students’ behavior in response to a major life event such as the pandemic.

Social class is known to be an important factor in wellbeing and in physical and mental health and is typically assessed through a joint consideration of level of education, income, and occupational prestige (Loignon and Woehr, 2018). Here, social class was measured by means of a 5-point scale question from working class (1) to upper class (5).

The BMI was defined as $\frac{w\,e\,i\,g\,h\,t\,\left(k\,g\right)}{h\,e\,i\,g\,h\,t^{2}\,\left(m^{2}\right)}$, which is an index, correlating with body fat content (Manson et al., 1987). In this study, underweight and overweight were incorporated in a single category, being associated with BMI < 18.5 and BMI > 30, respectively (World Health Organization [WHO], 1997). Students’ height and weight were recorded, and BMI was estimated based on the above discussion. BMI was included as a factor because being overweight or underweight might have a association with health.

For instance, although overweight individuals are more likely to suffer from stress and chronic conditions such as joint pain, heart disease, and cancers (Trakas et al., 1999), being underweight is associated with poor nutrition and infertility (Tjepkema, 2006). In addition, underweight individuals may have undiagnosed diseases and often have an increased risk of mortality (Kelly et al., 2010). BMI has previously been shown to be related to quality of life (Ford et al., 2001), which has shown that in the US, higher obesity (BMI > 30) is associated with lower health-related quality of life.

Finally, general health was measured based on a single item as “health in general” on a 5-point response scale from poor to excellent (The Centers for Disease Control and Prevention’s [CDC], 2018).

## Materials and methods

As the Tobit model with application of B-spline was used for the data analysis, the following explanation outlines the process. For the Tobit model, there is a concentration of observations at limit boundaries and a great possibility of having a negative or positive deviation from those values (Tobin, 1958). In other words, although it is expected much of the respondents selected the first or last alternatives, they had some positive or negative deviations from the extreme points. As a result, the upper and lower limits are expected to vary across all respondents; that is why the Tobit model was used for the analysis in this study.

With *P*(*Y* = *L*) = *P*(Y*≤*L*) and *P*(*Y* = *U*) = *P*(Y*≥*U*), the likelihood of Tobit model is:


(1) $$\prod_{y_{i}=L}\Phi \,\left(\frac{L-\mu_{i}^{*}}{\sigma_{i}}\right)\,\prod_{L<y_{i}<U}\frac{1}{\sigma_{i}}\,\Phi \,\left(\frac{y_{i}-\mu_{i}^{*}}{\sigma_{i}}\right)\,\prod_{y_{i}=U}\Phi \,\left(\frac{-\left(U-\mu_{i}^{*}\right)}{\sigma_{i}}\right)$$

where Φ and ϕ are Gaussian cumulative distribution function (CDF) and probability density function (PDF), respectively. As can be seen from Eq. 1, the likelihood function comprises a mixture of two discrete parts and the continuous in the middle. In addition, $\mu_{i}^{*}=x_{i}^{⊤}\,\beta +\varepsilon_{i},\text{where}\,\varepsilon_{i}∼N\,\left(0,\sigma^{2}\right).$ It is clear that while getting the log likelihood (*LL*) of Eq. 1, the final *LL* is the sum of three expressions across all observations: those at the limits of *U* and *L*, and those for < *y*_*i*_ < *U*.

As there are no values greater or lower than upper bounds and lower values, the model is called the standard Tobit model, or uncensored Tobit.

The model is solved by means of iteratively reweighted least square (IRLS), or maximization by means of deviance minimization. Now, the linear predictor of η=*g*(θ) is written as:


(2) $$\eta =\sum_{p=1}^{P}\beta_{p}\,x_{p}$$

where *p* and β are the number and parameters to be estimated.

For the above model based on the combination of all explanatory variables in a single matrix, and for iteration *a*, it could be written as: η^(*a*)^ = *X_VLM_β^a^*, and *X*_*VLM*_ is given by *X*_*VLM*_ = *X*⊗*I*_*M*_ (Yee, 2010), where *X*_*VLM*_ is formed using the Kronecker process, ⊗, to modify the *X* matrices.

The estimation of model parameters, based on the IRLS, is by creation of matrices of transformed response as $z^{\left(n\right)}=\eta_{i}^{\left(n\right)}+{\left(W_{i}\right)}^{{\left(n\right)}^{-1}}\,u_{i}^{\left(n\right)}$, Where ${\left(u_{i}\right)}_{j}=\frac{\partial \ell_{i}}{\partial \eta_{j}}$ is the score vector for j*th* element, and ${\left(W_{i}\right)}_{j\,k}=\frac{-\partial^{2}\ell_{i}}{\partial \eta_{j}\,\partial \eta_{K}}$. (*W*_*i*_)_*jk*_ measures the amount of information each observation carries. Then, the transformed or working response, *z*, could also be written as:


(3) $$z_{n-1}=\beta_{n}\,X_{V\,L\,M}+\varepsilon_{n-1}$$

The generalized least square (GLS) system of equations is converted into the ordinary least square (OLS), by pre-multiplying both sides of Eq. 3 by the Cholesky decomposition for standardizing the error terms and removing the correlations across them. The Cholesky decomposition is used to obtain the *U* matrix, which is the square root of the weight, where the weight is *W* = *U*^⊤^ *U*. Now, the matrix of *U* is used for obtaining the OLS by multiplication of the left- and right-hand sides of Eq. 3 by *U*. So, Eq. 3 can be written as:


(4) $$z_{a-1}^{**}=X_{V\,L\,M_{a-1}}^{**}\,\beta_{a}+\varepsilon_{a-1}^{**}$$

Now, the above equation can be solved by the OLS.

The process could be summarized as, first, implementing the B-spline basis matrix for a polynomial spline due to nonlinearity of few observations. After creating B-splines for few observations, the implemented model is similar to the standard model.

A few points need to be clarified regarding the implementation of the B-spline.

Although the boundary knots are assigned by γ_0_ = *min*(*x*)andγ_*k* + 1_ = *max*(*x*), the internal knots are set by B-spline. One of the advantages of B-spline is that it has the minimal support, highlighting that overlap between various splines is minimal.

Consider the boundary knots and interior knots of γ_1_,…,γ_*K*_. Now, 2Q more knots are augmented, so we have *T* = (*T*_*s* = 1_,…,*T*_*s* = *K* + 2*Q*_)^⊤^ satisfying the expressions of *T*_1_ = ⋯ = *T*_*Q*_ = γ_0_, *T*_*k* + *Q* + 1_ = … = *T*_*k* + 2*Q*_ = γ_*k* + 1_.

Now, the *s*th B-spline basis function of order *q* (refer De Boor, 2001), recursively for knot sequence *T* is given as:


(5) $$\begin{array}{ll} 1).\text{ First for }s & =1,\ldots ,k+2Q-1,B_{s,1}\left(x\right)\\ & =\{\begin{array}{cc} 1, & T_{s}\leq x\leq T_{s+1}\\ 0, & otherwise \end{array} \end{array}$$

(6) $$\begin{array}{l} 2).\text{ Afterward, for }s=1,\ldots ,k+2Q-q,\text{ where }q>1\\ B_{s,q}\left(x\right)=\varphi_{s,q}B_{s,q-1}\left(x\right)+\left(1-\varphi_{s+1,q}\right)B_{s+1,q-1}\left(x\right) \end{array}$$

where for Eq. 5, $\varphi_{s,q}\equiv \frac{x-T_{s}}{T_{s+q-1}\,T_{s}}$, whereas for the denominator we have *T*_*s* + *q*−1_ = *T*_*s*_. In addition, we have φ_*s*,*q*_≡0, in case of *T*_*s* + *q*−1_ = *T*_*s*_. Also (1φ_*s* + 1,*q*_) in Eq. 5 is $\frac{T_{s+q}-x}{T_{s+q}-T_{s+1}}$.

So, for instance, if we have 3 knots for DF = 1, the two boundary knots are used twice, whereas other knots for *s* are created for a single time and would be ordered and used for the B-spline. In addition, from the above equation, it is clear that for estimating the B-spline, knots, DF, and vector of observations are needed. Here, only a single parameter of being worry due to COVID-19 was modeled based on B-spline. In addition, the subjective health is the response of the model.

Finally, a question might be raised regarding the appropriateness of the Gaussian distribution assumption for Likert data, similar to those collected in our survey. First, the respondents were able to choose any continuous value within the interval. Second, even if we considered discrete values only, it has been established that there is consistent support for treating this Likert type of variable as approximately continuous (Norman, 2010).

## Results

The results are presented in Table 2; first, for those predictors that their main effects were considered only, and then for interaction terms.

**TABLE 2:** Factors associated with subjective wellbeing.

Coefficients	Estimate	SE	Pr ( > | z|)
(Intercept):1	4.63	0.416	<0.005
(Intercept):2	0.06	0.016	<0.005
Gender	−0.39	0.150	0.009
Mother’s education	−0.04	0.025	0.1
Feeling irritability due to COVID-19	0.002	0.004	0.5
Feeling guilty due to COVID-19	−0.002	0.001	0.1
Screen hours	−0.02	0.024	0.5
Students’ relative family income	0.21	0.089	0.02
Limiting exercise at gym due to COVID-19	0.08	0.050	0.1
BMI	−0.74	0.138	<0.005
Feeling afraid due to COVID-19	−0.004	0.001	<0.005
Social class of students	0.15	0.028	<0.005
Feeling sad due to COVID-19	−0.003	0.002	0.1
Student education achievement	0.09	0.057	0.1
Limiting exercise at home due to COVID-19	−0.18	0.047	<0.005
Limiting outdoor activities due to COVID-19	−0.07	0.020	<0.005
Gender × mother’s education	0.06	0.031	0.06
Screen hours × relative family income	−0.02	0.010	0.06
Feeling irritability due to COVID-19 × limiting exercise at gym due to COVID-19	−0.001	0.001	0.08
BMI × being afraid due to COVID-19	0.01	0.002	0.03
Being sad due to COVID-19 × limiting exercise at home	0.001	0.001	0.08
**B-spline**			
(Feeling worry due to COVID-19)1	−0.09	0.224	0.7
(Feeling worry due to COVID-19)2	−0.44	0.152	<0.005
(Feeling worry due to COVID-19)3	0.10	0.152	0.5

### Main effects

The associations between few variables and subjective health are based on only main effects. Those include various negative feelings, social status, education, and cautionary behavior, which the next few paragraphs are outlined.

Although negative feelings of guilt, ${\hat{\beta }}_{G\,u\,i\,l\,t\,y}=-0.002$, was found to be associated with a lower subjective health, the results highlighted that the graduate students, compared with the undergraduate students, are associated with better subjective health. Students’ higher social class and educational achievement are all associated with higher subjective wellbeings due to possible confounding factors, which are not recorded at the time of data collection.

### Interaction terms

Here, all pairwise interaction terms were considered to see if the associations of variables and subjective health are stable or multiplicative.

#### Female × mother’s education

In the current study, we found that the association between gender and the subjective health varies based on mother’s educations. For instance, the result of interaction term highlighted that while female students’ ${\hat{\beta }}_{G\,e\,n\,d\,e\,r}=-0.39$ are associated with lower subjective health, the impact is mitigated with increased level of mother’s education, ${\hat{\beta }}_{m\,o\,t\,h\,e\,r^{\prime }\,s\,e\,d\,u\,c\,a\,t\,i\,o\,n}=-0.04,{\hat{\beta }}_{i\,n\,t\,e\,r\,a\,c\,t\,i\,o\,n}=0.06$.

#### Screen hours × relative family income

The results highlighted that while students’ sedentary behavior such as screen time is negatively associated with the subjective health ${\hat{\beta }}_{S\,c\,r\,e\,e\,n\,h\,o\,u\,r\,s}=-0.02$, that variable is in tandem, with the magnitude of the contributory impact of family income is much higher ${\hat{\beta }}_{F\,a\,m\,i\,l\,y\,i\,n\,c\,o\,m\,e}=0.21$, compared with ${\hat{\beta }}_{I\,n\,t\,e\,r\,a\,c\,t\,i\,o\,n}=-0.02$.

#### Limit exercise at home × feeling sad

Both feeling sad and limiting exercise at home associate with a lower subjective health, and when a higher slope is related to limiting exercise at home, ${\hat{\beta }}_{L\,i\,m\,i\,t\,e\,x\,e\,r\,c\,i\,s\,e\,a\,t\,h\,o\,m\,e}=-0.18$, compared with ${\hat{\beta }}_{F\,e\,e\,l\,i\,n\,g\,s\,a\,d}=-0.003$ and the interaction term ${\hat{\beta }}_{I\,n\,t\,e\,r\,a\,c\,t\,i\,o\,n\,t\,e\,r\,m}=0.001$.

Irrational behavior like limiting exercise at home was considered in this study to verify if due to possible impact of various emotions, students take extreme behavior, which is irrelevant to being infected with the virus. In addition, with modeling behavioral approach, it is important to consider its psychological forces that might be associated with those behaviors.

#### Limiting exercising at gym × feeling irritable

Besides limiting exercise at home, another physical activity being considered was doing exercise at gym during the pandemic. Here, the interaction of doing exercise at gym and feeling irritable was considered. The results highlight that the association between screen time, ${\hat{\beta }}_{S\,c\,r\,e\,e\,n\,t\,i\,m\,e}=-0.02$ and subjective health varies based on the degree of limiting exercise at gym, ${\hat{\beta }}_{L\,i\,m\,i\,t\,e\,x\,e\,r\,c\,i\,s\,e\,a\,t\,g\,y\,m}=0.08$. In other words, the negative association between feeling irritable and subjective health decreases by limiting exercise at gym with a higher degree.

Comparing limiting exercise at home and gym during the pandemic highlights that although limiting exercise at home is associated with a poorer perceived health, limiting exercise at gym is positively associated with the general health of students.

#### Body mass index × feeling afraid

Our finding highlights the association between feeling afraid due to COVID-19 and subjective health varies based on students with various BMI. In other words, both overweight or underweight, ${\hat{\beta }}_{B\,M\,I}=-0.74,$ and higher emotion of being afraid, ${\hat{\beta }}_{A\,f\,r\,a\,i\,d}=-0.004$, associate with the poorer subjective health, whereas they are interacting, ${\hat{\beta }}_{i\,n\,t\,e\,r\,a\,c\,t\,i\,o\,n}=0.01$. Therefore, based on the above finding, it is clear that the impact of BMI is much higher than the impact of feeling afraid on the perceived quality of health. In addition, the impact of BMI would be exacerbated by an increase in the level of being afraid due to COVID-19.

**FIGURE 1:**
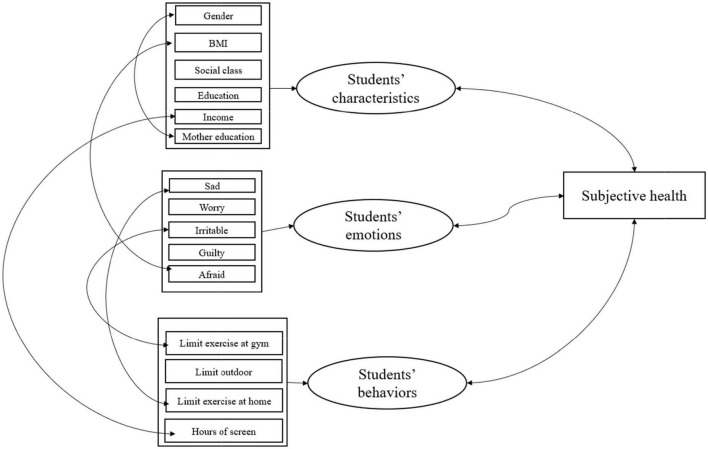
Conceptual model of interrelationship of subjective health and other predictors, and arrows in LHS and RHS are interaction and associations, respectively.

Finally, the smoothing of a single predictor resulted in a slight enhancement of the model fit, Akaike information criterion (AIC)=6,520 and loglik=−3,236 compared with the standard model with AIC=6,527 and loglik=−3,242. In summary, as can be seen from Figure 1, the construct of the subjective health is shown at the core of the framework. Based on the figure, wellbeing has been seen as an encompassing construct, comprising individual characteristics, emotions, and behaviors. In addition, the impacts of majority of predictors are not stable but multiplicative.

## Discussion

In this study, we analyzed survey data from college students to explore possible associations between subjective health during the time of pandemic and various students’ characteristics. Given the centrality of subjective perceptions of health to physical health (Pinquart and Sörensen, 2003) and other indices of quality of life (Bullinger, 1997), this study sought to identify factors associated with students’ subjective health. Especially, we challenged the assumption of studies in the literature, which mainly considered the additive relationship between subjective health and various predictors (e.g., refer Kahneman and Krueger, 2006).

The findings revealed that while interpersonal students’ factors such as relative family income, social class, and mother’s education help to buffer against the lower subjective health, higher screen hours, negative feelings, and so-called cautionary behaviors such as limiting exercise at home contribute to the lower subjective health.

Here, only the associations of few variables and subjective health were found to be stable. Those include students’ interpersonal factors of social class and educations, students’ negative emotions of being guilty, and cautionary behavior of limiting outdoor activities due to COVID-19.

Negative feeling of guilt was found to be associated with a lower subjective health. Implication of being guilty on health was evaluated in a previous study, and it was found that higher level of guilt is associated with poorer mental health (vanOyen Witvliet et al., 2001). However, those studies considered guilt in a general sense, whereas in this study guilt was due to COVID-19. In general, the results are in line with the previous study, which found that various negative feelings are associated with the poorer psychological wellbeing (Jirojwong and Manderson, 2001).

We found that higher social class is associated with higher subjective health. Social class has been found as an important factor in mental and physical health of upper class in relation with lower class (Chen and Miller, 2013); the impact was linked to the fact that lower-social-class individuals have higher negative feelings such as stress because they have fewer resources to control the environment’s confounders, and thus they experience a higher amount of helplessness and uncertainty (Pepper and Nettle, 2017).

We found that higher level of education is associated with a lower subjective health. Similarly, higher education has been found to be associated with a better mental health (Cairney and Krause, 2005). In another study, the relationship between education and subjective quality of life was evaluated (Ross and Van Willigen, 1997); it was found that well-educated individuals experience lower level of dissatisfaction due to a higher personal control. In the aforementioned study, variety of indicators were used for evaluation of the subjective quality of life, including depression, anxiety, anger, and pain.

It should be noted that during the pandemic, outdoor activity is not as risk-free, arguably, as exercising at home. The results highlighted that both limiting outdoor activities correspond to lower subjective health ratings, which accord with literature attesting to the importance of outdoor activities in promoting physical activity and health improvement (Kline et al., 2011).

On other hand, we found that the associations between the majority of factors and subjective health are not additive. For instance, it was found that the association between BMI and subjective health varies based on mothers’ education of students, or associations of irrational cautionary behavior of limiting exercise at home varies based on students’ sadness due to the pandemic.

The disparities in screen-based time indicated that students with lower-income families while interacting with screen time, they are associated with a poorer health. The results highlight the importance of family contextual factors on the wellbeing of students during the pandemic. It is expected that lower family income to be linked with parenting behaviors and cognition, which itself shape students’ attitudes.

A primary finding that concerned with demographic and individual characteristics showed that higher amounts of education, social class, and income are associated with improved subjective wellbeing, even during the pandemic. This may be explained by their likely implications for better life prospects, greater sense of control, and better understanding of problems in life.

We found that while gender is interacting with mother’s education, it impacts the subjective health. Specifically, we found that while women are experiencing lower subjective health, the association with subjective health varies based on mother’s education. Sex-specific variations in response to stress have been reported in a previous study (Verma et al., 2011), highlighting that chronic pain, depression, and anxiety disorders are more prevalent in women (Lundberg, 2005).

Behaviors reported in the survey could be categorized as rational, such as limiting exercise at gym, and irrational, such as limiting exercise at home during the pandemic. The results highlighted that while the irrational behavior associates with a lower subjective wellbeing, remarkably, rational behaviors such as limiting exercise at gym associates with a higher subjective wellbeing. That might be due confounding associations between taking various actions and the perceived wellbeing of students.

As there is no associated risk of being infected by doing exercise at home, it is worth discussing what might cause that behavior. That is especially important as that behavior was found to be negatively associated with subjective health. Although from outsiders’ perspective that could be due to lack of motivation, for students’ perspective that could be explained as emotional excuses. We speculated that the students’ judgment is impaired due to negative feelings experienced due to the pandemic, especially feeling of being sad, due to its interaction term.

We judged the translated impact of sad as an “excuse” since there is no cause to blame for this behavior. In other words, fear of extreme threat to the pandemic, translated into emotional excuses, or overreaction, which prevents students from doing even at-home exercise. As the interaction terms of sad was found to impact exercising at home, students under emotional conditions and during critical circumstances should be reminded especially about the importance of regular physical activity as they are vital for good mental and physical health (Katzmarzyk et al., 2019).

A special attention should be directed toward clarification to address irrational behaviors, e.g., limiting exercise at home. Students also should be learnt the ability to control the intensity of the emotional response such as feeling sad, as those emotions were found to be important predictors of subjective health. Policies should also be stratified before implementations based on various students’ characteristics, e.g., mother’s education or BMI, e.g., obese, as it was found that there are interactive relationships between those factors and subjective health.

Emotions undermine the ability to conform to the mind normality, so students should be reminded about the plausible rational and irrational behavior due to the negative stimuli and their implications not only on the perceived or subjective health but also on the objective health and its psychological effects. Our results also confirmed the multiplicative relationships of irrational behaviors and negative emotions on the subjective health.

As expected, reports of more screen hours were found to be negatively associated with subjective wellbeing. The impact of higher screen hours could be linked to increased dissatisfaction and deprivation caused by the pandemic. However, it is worth mentioning that the screen time impact was mitigated by limiting exercise at gym and more importantly by higher income of family.

Despite the importance of studying the multiplicative impacts of screen time and doing exercise at gym, the majority of previous studies considered only their additive impacts. For instance, the association between screen time and subjective well_being (SWB) is well-established (García-Hermoso et al., 2020). However, in this study instead of the main effects of limiting exercise at gym and hours in front of screen, their interaction terms were incorporated. It should be noted that although a previous study considered exercise and screen time on the mental and general health, their main effects were used only by means of descriptive summary (Colley et al., 2020).

We noted that as the results emerge from unique survey data, our results are necessarily limited to the population studied (i.e., college students) and by the self-report methodology, which may have especially challenged respondents in requiring them to relay the amount of time spent on different activities within the past 24 h. The data also constrain inferences of cause-and-effect relationships, especially among the psychological and behavioral factors. In addition, BMI measurement was based on students’ self-reported heights and weights. However, it has been found that both men and women may have overestimated their height and/or underestimated their weight (Danubio et al., 2008), which might bias the BMI. For future studies, having interviews with students while measuring their height and weight and linking those values with BMI could confirm the obtained results.

Nevertheless, the study provides novel information about associations that are candidates for causal relationships, a first step toward information that could usefully inform policy during times of unprecedented turmoil as during the pandemic.

## Conclusion

Subjective health assessment is important as it is expected to be in line with the objective health status. In addition, the evaluation of general health is significant as the general health is not only a matter of physical health, but also it is an integration of the health of body and mind. While we found that the impacts of some predictors are additive, the impacts of majority of variables were found to be multiplicative. Considering the complex relationship between variables and subjective health is especially important as effective policies could be directed toward those associations to enhance the health and wellbeing of individuals.

In summary, our results highlighted the interactive relationship between various behaviors and negative emotions and the subjective wellbeing of students. The future studies are recommended to account for the complex relationship between various behaviors and emotions by considering their interactive relationship while studying subjective health.

## Data availability statement

The original contributions presented in the study are publicly available. This data can be found here: doi: 10.1371/journal.pone.0245327.

## Ethics statement

Ethical review and approval was not required for the study on human participants in accordance with the local legislation and institutional requirements. Written informed consent from the patients/participants or patients/participants legal guardian/next of kin was not required to participate in this study in accordance with the national legislation and the institutional requirements.

## Author contributions

The author confirms being the sole contributor of this work and has approved it for publication.
